# Proteomic changes in rat hippocampus and adrenals following short-term sleep deprivation

**DOI:** 10.1186/1477-5956-6-14

**Published:** 2008-05-22

**Authors:** Jean-Etienne Poirrier, François Guillonneau, Jenny Renaut, Kjell Sergeant, Andre Luxen, Pierre Maquet, Pierre Leprince

**Affiliations:** 1 Center for Cellular and Molecular Neurobiology, University of Liege, CHU B36, Avenue de l'Hopital 1, B – 4000 Liege, Belgium; 2 Cyclotron Research Center, University of Liege, B30, Allee du 6 Aout 8, B-4000 Liege, Belgium; 3 Laboratory of Mass spectrometry, University of Liege, B6c, Allee de la Chimie 3, B-4000 Liege, Belgium; 4 Centre de Recherche Public-Gabriel Lippmann, Department Environment and Agrobiotechnologies, Rue du Brill 41, L-4422 Belvaux, Luxembourg

## Abstract

**Background:**

To identify the biochemical changes induced by sleep deprivation at a proteomic level, we compared the hippocampal proteome of rats either after 4 hours of sleep or sleep deprivation obtained by gentle handling. Because sleep deprivation might induce some stress, we also analyzed proteomic changes in rat adrenals in the same conditions. After sleep deprivation, proteins from both tissues were extracted and subjected to 2D-DIGE analysis followed by protein identification through mass spectrometry and database search.

**Results:**

In the hippocampus, 87 spots showed significant variation between sleep and sleep deprivation, with more proteins showing higher abundance in the latter case. Of these, 16 proteins were present in sufficient amount for a sequencing attempt and among the 12 identified proteins, inferred affected cellular functions include cell metabolism, energy pathways, transport and vesicle trafficking, cytoskeleton and protein processing. Although we did not observe classical, macroscopic effect of stress in sleep-deprived rats, 47 protein spots showed significant variation in adrenal tissue between sleep and sleep deprivation, with more proteins showing higher abundance following sleep. Of these, 16 proteins were also present in sufficient amount for a sequencing attempt and among the 13 identified proteins, the most relevant cellular function that was affected was cell metabolism.

**Conclusion:**

At a proteomic level, short term sleep deprivation is characterized by a higher expression of some proteins in the hippocampus and a lower abundance of other proteins in the adrenals (compared to normal sleep control). Altogether, this could indicate a general activation of a number of cellular mechanisms involved in the maintenance of wakefulness and in increased energy expenditure during sleep deprivation. These findings are relevant to suggested functions of sleep like energy repletion and the restoration of molecular stocks or a more global homeostasis of synaptic processes.

## Background

Sleep is present in all vertebrate species studied so far suggesting that it might underpin one or several vital functions [1,2]. Sleep has been associated with functions as diverse as thermoregulation [3], energy conservation [4], immune defense [5], tissue restoration [6] and brain plasticity [7,8].

One way to approach the functions of sleep is to assess the effects of sleep deprivation on behavioral and physiological responses at the organism, tissue and cellular levels. Several studies addressing gene transcription through the sleep-wake cycle identified a number of genes that are differentially expressed during wakefulness and sleep (for reviews, see [9,10]). Physiological changes in cellular properties are eventually derived from gene transcription followed by gene translation and protein synthesis. In consequence, the characterization of protein changes that contribute to the cellular phenotype is an indispensable complement to genomic studies in understanding the link between cellular activity and behavior.

To our knowledge, at the present time, only one proteomic study of the effect of sleep deprivation has been published. Using classical 2D-gel electrophoresis, Basheer et al. studied levels of protein expression in the rat basal forebrain after 6 hours of sleep deprivation by gentle handling [11]. In this analysis, 89 spots showed more than a two-fold difference between 6 hours sleep-deprived rats and undisturbed sleeping controls. The fact that several identified proteins in their study either belong to the cytoskeleton or are closely associated with synaptic function suggests that changes in synaptic transmission or plasticity may occur after 6 hours of sleep deprivation in a wake-promoting area of the rat basal forebrain.

Since the hippocampus plays an important role in spatial memory [12], in humans [13] as well as in rodents [14] and sleep deprivation is known to disturb the memory consolidation process [15], our objective was to identify changes in protein levels occurring in the rat hippocampus after either a short (4 hours) period of sleep or of total sleep deprivation by gentle handling. Since the hippocampus is also a target of stress hormones [16], we measured stress by classical stress indices [17] (stomach ulceration, adrenal hypertrophy and body weight loss). We also looked at the proteomic changes in the rat adrenals after 4 hours sleep deprivation since adrenals are physiologically affected by sleep deprivation [18].

## Results

### Sleep deprivation

**Figure 1:**
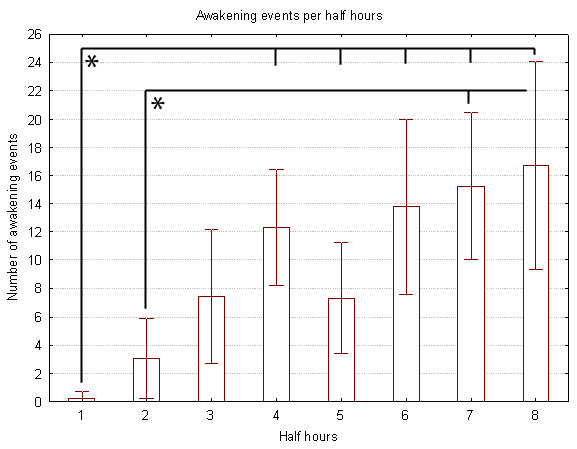
**Number of interventions that were required to prevent the occurrence of sleep for consecutive 30-min intervals**. Significant differences were observed between intervals 1–4 and 2-7-8.

The activity of rats during 4 hours at the beginning of the light period was behaviorally scored. The mean duration of behaviorally scored sleep in undisturbed rats was 144.6 ± 56.8 minutes. Sleep-deprived rats never slept during the 4 hours of sleep deprivation. Undisturbed rats were not awoken while sleep deprived rats were awoken 80.8 ± 22.5 times during the 4 hours. Figure 1 shows, for consecutive 30-min intervals, the number of interventions which were required to prevent the occurrence of sleep. The number of interventions increased progressively during the first 2 hours and then remained at a high level (except for the 5th interval, i.e. the period from 2h00 to 2h30). A one-way ANOVA for repeated measures revealed a significant effect of the factor "interval" (p < 0.05). Posthoc test (Tukey test) revealed significant increase in the number of interventions between the first half hour and the fourth to eighth ones and between the second half hour and the seventh and eighth intervals.

### Stress indices

Rats were sacrificed immediately following the 4 hours of sleep or sleep-deprivation. The whole brain, hippocampus, stomach and adrenals were removed, weighed and stored at -80°C. The stomach was opened and, after careful observation, we were unable to find any ulceration. Adrenal weight was compared between sleep-deprived and undisturbed rats and no significant difference was found (Kruskal-Wallis, p-value > 0.05). Body weight also did not significantly change between the two groups (Kruskal-Wallis, p-value > 0.05).

### Hippocampal proteins profiling by 2D-DIGE and identification of regulated proteins in undisturbed and sleep-deprived rats

**Figure 2:**
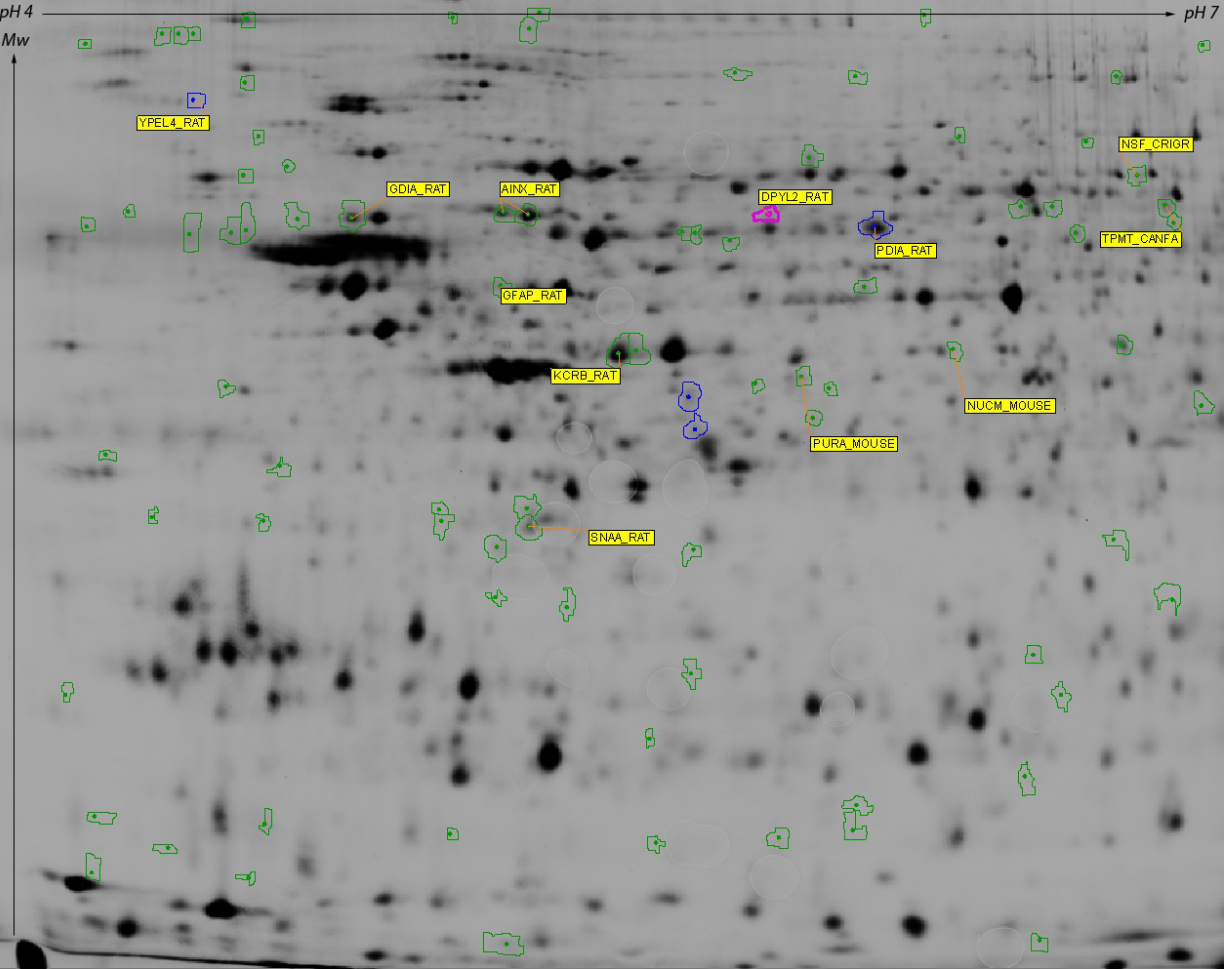
**Representative bi-dimensional maps of the proteome of sleep-deprived rat hippocampi between pH 4 and 7**. Delimited spots correspond to proteins of interest that were picked for mass spectrometry identification. ID of identified proteins from table 1 are indicated in yellow boxes.

Hippocampal proteins were extracted, labeled with either Cy3 or Cy5 CyDye and separated by isoelectric focusing between pH 4 and 7 (first dimension). After reduction and alkylation, proteins were then separated following their molecular weight in a second dimension electrophoresis. After scanning the gels, the images of the 2D distribution of hippocampal proteins were analysed. Figure 2 shows a representative bi-dimensional map of the proteome of sleep-deprived rat hippocampi. CyDyes protein labeling combined with automatic spot detection enables visualization of about 4000 individual spots on a 2-D gel. The protein pattern from 4 h sleep-deprived rat hippocampi (n = 6) was globally very similar to that of undisturbed rat (n = 6). However, after matching spots, 87 spots showed significant variation between sleep and sleep deprivation: 64 spots were upregulated by a factor 1.2 to 2.94 following 4 h sleep deprivation, while 23 spots were downregulated by a factor 1.2 to 2.74. From the 16 selected spots sent to the MALDI-TOF mass spectrometry, we unambiguously identified 12 proteins. Their name, accession number, change in relative abundance and other MS data are summarized in table 1.

**Table 1:** Hippocampal proteins whose abundance differs between conditions with protein ID from Swiss Prot database, analysis and identification information.

**Master #**	**Protein ID**	**Name**	**Function**	**Average ratio**	**p-value**	**pI**	**Mw**	**Mowse score**
1169	AINX_RAT	*α*-internexin	Y	-1.52	0.000082	5.2	56253	108
1166	DPYL2_RAT	Dihydropyrimidinase-related protein 2	D	-1.64	0.012	5.95	62638	100
1192	GDIA_RAT	Rab GDI *α*	G	-1.31	0.045	5	51074	100
1489	GFAP_RAT	Glial fibrillary acidic protein	Y	-1.35	0.04	5.35	49970	90
1781	KCRB_RAT	Creatine kinase type B	M	-1.26	0.045	5.33	42970	236
971	NSF_MOUSE	Vesicle fusing ATPase	T	-1.33	0.0067	6.52	83083	68
1753	NDUS2_MOUSE	NADH ubiquinone oxidoreductase	M	-1.22	0.012	6.52	52991	69
1230	PDIA3_RAT	Protein disulfide isomerase A3	C	1.83	0.033	5.88	57044	100
1869	PURA_MOUSE	Transcriptional activator protein Pur-*α*	A	-1.23	0.025	6.07	34976	54
2462	SNAA_RAT	*α*-soluble NSF attachment protein	T	-1.37	0.017	5.3	33627	107
1131	TPMT_CANFA	Thiopurine S-methyltransferase	C	-1.42	0.0071	6.46	28752	87
532	YPEL4_RAT	Protein yippee-like 4	?	1.53	0.036	8.42	14762	44

In the Function column, A = transcriptional regulation, C = protein translation, folding and degradation, D = development, G = cell signalling, M = cell metabolism and energy, S = stress, chaperone, T = transport and vesicle trafficking, Y = cytoskeleton. In the Average ratio column, a negative number corresponds to a protein more abundant after 4 hours of sleep deprivation than 4 hours of sleep. The average ratio represents the ratio between the average protein abundances in the different conditions (n = 6), as computed by the DeCyder software. Other abbreviations: pI = identified isoelectric point ; Mw = identified molecular weight. Mowse score as defined by Pappin et al. [62]

### Adrenals proteins profiling by 2D-DIGE and identification of regulated proteins in undisturbed and sleep-deprived rats

We next examined the effect on the adrenals proteome following the exposure of rats to 4 h of sleep deprivation. As for the hippocampal proteins, the protein pattern from 4 h sleep-deprived rat adrenals (n = 4) was globally very similar to that of undisturbed rat adrenals (n = 4). Fifty-seven spots were found to display a significant variation in relative abundance after 4 hours of sleep deprivation, compared to undisturbed rats: 18 spots were more abundant (factor of 1.21 to 1.62), whilst 39 spots were less abundant by a factor of 1.20 to 4.72. From the 16 selected spots sent to the MALDI-TOF mass spectrometry, we unambiguously identified 13 proteins. Their name, accession number, change in relative abundance and other MS data are summarized in table 2.

**Table 2:** Adrenal proteins whose abundance differs in the two conditions (n = 4) with protein ID from Swiss Prot database, analysis and identification information.

**Master #**	**Protein ID**	**Name**	**Function**	**Average ratio**	**p-value**	**pI**	**Mw**	**Mowse score**
1372	G6PD_RAT	Glucose-6-phosphate 1-dehydrogenase	M	-1.49	0.0097	5.97	59375	103
2107	A1M_RAT	*α*-1-macroglobulin	M	-1.27	0.018	6.42	167125	166
2744	CLIC4_RAT	Chloride intracellular channel protein 4	T	-1.25	0.046	5.93	28502	-
1525	Q4KLG7_RAT	Serine hydroxymethyltransferase 1	M	1.23	0.047	6.96	38106	241
2912	STAR_RAT	Steroidogenic acute regulatory protein	A	1.24	0.016	8.93	31501	141
1151	ALBU_RAT	Serum albumin	M	1.27	0.041	5.8	65916	45
1032	GRP78_RAT	Heat shock 70kD protein 5	T	1.28	0.034	5.01	70475	302
1438	SYDC_RAT	Aspartyl-tRNA synthetase	C	1.31	0.0042	6.02	57126	348
2038	NDUAA_RAT	NADH dehydrogenase 1 *α*	M	1.71	0.048	5.96	36843	98
1503	AL9A1_RAT	Aldehyde dehydrogenase family 9, subfamily A1	M	1.79	0.027	7.94	54327	236
1551	ALDH2_RAT	Aldehyde dehydrogenase precursor	M	1.93	0.031	5.69	54368	344
1361	CES3_RAT	Carboxylesterase	M	2.05	0.029	6	60054	180
1537	ALDH2_RAT	Aldehyde dehydrogenase precursor	M	2.32	0.021	5.69	54368	482

All proteins were identified by PMF except one without Mowse score which was identified by MS/MS. Same notes as in table 1.

### Western blots

To confirm the validity of the quantitative data obtained in our proteomic analysis, we further performed selected western blots analysis on several samples to verify that our 2D-DIGE analysis indeed reflected true changes in tissue protein expression.

**Figure 3:**
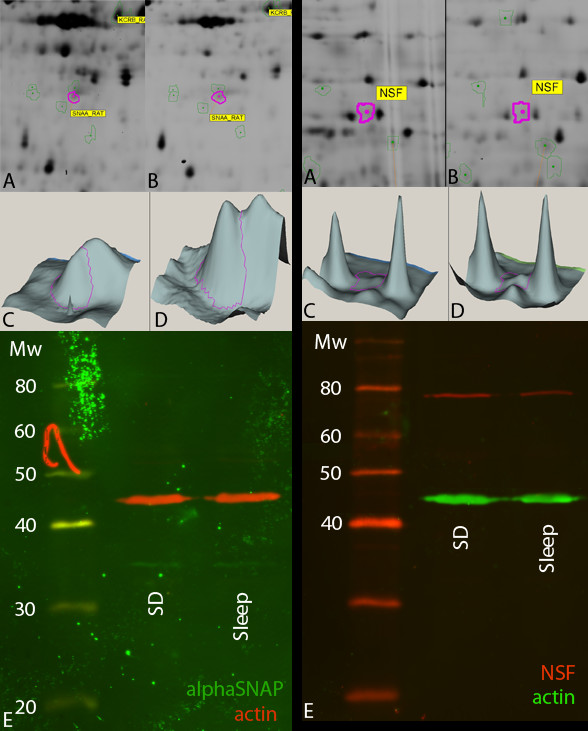
**Representative 2D gel regions (A, B), 3D visualization of the spot contour in purple (C, D) and western blots (E) for NSF (left set of images) and alpha-SNAP (right set of images)**. Average ratio from 2D-DIGE analysis of the spot containing NSF was -1.33 (n = 9); average band volume ratio from western blot analysis was -1.28 (n = 3). Average ratio from 2D-DIGE analysis of the spot containing alpha-SNAP was -1.37 (n = 9); average band volume ratio from western blot analysis was -1.15 (n = 3). For all western blots, the variations in sample loading revealed by the actin signal is taken into account for the calculation of the average band volume ratio.

For the hippocampus, we exposed extracted proteins after separation by SDS-PAGE to antibodies directed against *α*-SNAP and NSF. After spot volume analysis, taking into account variations in sample loading revealed by the actin signal, the ratio of NSF between the sleep and sleep-deprivation conditions was -1.28 (similar to the value -1.33 from the DIGE analysis; figure 3). The ratio of *α*-SNAP between these two conditions was -1.15 (similar to the value -1.37 found in the DIGE analysis; figure 3).

**Figure 4:**
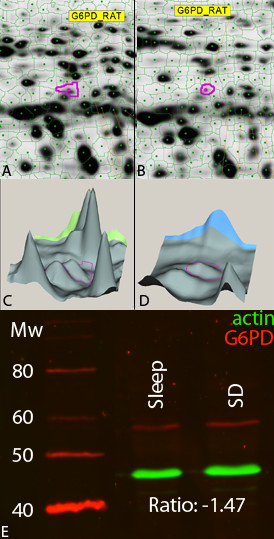
**Representative 2D gel regions (A, B), 3D visualization (C, D) and western blots (E) for Glucose-6-phosphate dehydrogenase**. Average ratio from 2D-DIGE analysis of the spot containing Glucose-6-phosphate dehydrogenase was -1.49 (n = 9); average band volume ratio from western blot analysis is -1.47 (n = 3). Variations in sample loading revealed by the actin signal is taken into account for the calculation of the average band volume ratio.

We separated adrenals proteins by 1D-SDS-PAGE and exposed them to an antibody directed against glucose-6-phosphate dehydrogenase. After incubation with an ECL Plex fluorescent secondary antibody and scanning, the presence and abundance of the protein was analyzed (figure 4). After spot volume analysis taking into account variations in sample loading revealed by the actin signal, the ratio of glucose-6-phosphate dehydrogenase between the sleep and sleep-deprivation conditions was -1.47 (similar to the value of -1.49 from the DIGE analysis).

## Discussion

In this work we were able to identify, through proteomic analysis, 31 proteins the abundance of which was modified in hippocampal or adrenal tissues after 4 hours of sleep deprivation by gentle handling as compared to a similar period of sleep. In the discussion below, proteins that are more expressed in rat left undisturbed have positive ratios while proteins that increase after sleep deprivation display negative ratios. Most of the identified proteins in hippocampi were more abundant after short term sleep deprivation than after normal sleep. This result is consistent with transcriptomic studies in which sleep deprivation induced the over-expression of selected mRNAs in the hippocampus [19]: most genes are consistently upregulated during/after waking and short-term sleep deprivation relative to sleep [9]. Surprisingly, most of the identified proteins in the adrenals were more abundant after normal sleep than after short term sleep deprivation. It should however be cautioned that the occurrence of higher protein levels in one behavioral state relative to another may not only reflect an increase in translation rate but also a reduced degradation of proteins, a post-translational modification of proteins or a combination of these events. 2D-DIGE, the technique employed in this work, while allowing the detection of subtle differences in protein properties or abundance, does not permit to distinguish between these possibilities.

By using an advanced quantitative multiplex fluorescence immunoblotting methodology, we were able to quantitatively confirm the differences in abundances of NSF and *α*-SNAP in the hippocampus and G6PD in the adrenals of rats.

### Identification of proteins in hippocampus of sleep-deprived rats

In the hippocampus of sleep-deprived rats, proteins showing a higher abundance participate in five main functions: cell metabolism, energy pathways, transport and vesicle trafficking, cytoskeleton and protein processing (translation, folding and degradation). Importantly, no protein related to stress response were identified in the hippocampus although cellular stress response genes were upregulated after 3–8 h of spontaneous waking [9].

All the functions attributed to proteins the abundance of which increases after sleep deprivation, require energy. An increase in the expression of genes related to energy metabolism has already been described in the literature [20]. Our results support the hypothesis of a fast adaptation of neurons and/or glial cells to the increased metabolic demand of wakefulness relative to sleep [20]. They are also consistent with an increase in energy requirement possibly related to a progressive increase in synaptic strength with prolonged wakefulness [21] (although none of the proteins identified here are the products of gene markers for synaptic potentiation [22]).

Accordingly, two central enzymes for cellular energy metabolism were identified. Creatine kinase (ratio of -1.26), a major enzyme of the phosphotransfer system in cells, plays a central role in energy transduction in tissues with large, fluctuating energy demands (such as skeletal muscle, heart, brain and spermatozoa) [23]. It acts in concert with other enzymatic systems to facilitate intracellular energetic communication (creatine kinase/phosphocreatine "spatial energy transport" system [23]) and is responsible for maintaining the phosphocreatine levels, an acute energy reserve [24]. Creatine kinase is a cytoplasmic protein also expressed in the mitochondrion whereas NADH ubiquinone oxidoreductase (aka. Complex I, ratio of -1.22) is only expressed in the latter. NADH ubiquinone oxidoreductase catalyzes the oxidation of NADH, the reduction of ubiquinone, and the translocation of 4H^+ ^across the coupling, inner-mitochondrial membrane eventually leading to ATP synthesis [25].

The increased abundance of Rab GDI *α *(ratio of -1.31), vesicle fusing ATPase (NSF) (ratio of -1.33) and the *α*-soluble NSF attachment protein (*α*-SNAP) (ratio of -1.37) in the hippocampus suggests an increased regulation of transport and vesicle trafficking, especially the docking and dissociation of vesicles to their target organelles. As proteins interacting with SNARE, *α*-SNAP and NSF play a central role in the specificity of the docking between a vesicle and its target membrane [26]. After vesicle docking, NSF will dissociate SNARE pairs and thus control the time and location of membrane fusion. Finally, Rab GDI *α *is an important factor in the control of Rab and thus the specificity of vesicular transport. By binding Rab, released after membrane fusion, Rab GDI prevents Rab from releasing its GDP molecule until it has interacted with appropriate proteins in the donor membrane [27,28]. This increased regulation of vesicle trafficking could underlie a general increase in synaptic activity reflecting the induced motor activity and alertness as well as a reaction to a stressful situation. Interestingly, several authors have found a related increase in gene transcription in the cerebral cortex of undisturbed rats (calcineurin, CaMKIV and the Rab family of genes, see [29,30]).

*α*-soluble NSF attachment protein is also the only protein from our study that is related to the *β*-soluble NSF attachment protein, found by Basheer et al. in the only other proteomic study of the effects of sleep deprivation published so far [11]. These two proteins are very similar in sequence and have the same functions in the cell. However, the abundance of the *α*-soluble NSF attachment protein is increased after sleep deprivation while the expression of the *β*-soluble NSF attachment protein is decreased after sleep deprivation. This discrepancy could be explained by the fact changes in cytoskeletal proteins after sleep deprivation could go in various directions depending on the precise need of synapses (since we use a different duration and study a different brain area than Basheer et al.).

Sleep deprivation increases at least two proteins related to cytoskeletal function, neural development and synaptic plasticity: *α*-internexin and GFAP. *α*-Internexin (a component of intermediate filaments, ratio of -1.52) is a marker of neuronal tissue and a critical protein for neuronal development [31]. Thus its increased abundance, after a short-term sleep deprivation in the rat hippocampus, may indicate the presence of new neurons or their morphological modification to fit a possible synaptic potentiation [21]. Its importance for axonal caliber and nerve conduction speed, along with its correlation with glutamate transporter levels in the energetically compromised post-injury brain [32] gives *α*-internexin a potential role in energy homeostasis. Other studies also found it upregulated after voluntary exercise [33] in the same way as expression of LTP-related genes increased during extensive exploration [34]. All in all, this higher abundance of *α*-internexin seems to be consistent with the homeostatic synaptic hypothesis [21]. Here, since the duration of the sleep deprivation episode is probably too short to elicit neuron proliferation, the change in *α*-internexin abundance would simply be due to post-translational modifications related to synaptic plasticity.

Glial fibrillary acidic protein (GFAP) (ratio of -1.35) is a major component of the glial astrocyte cytoskeleton and is generally used as a hallmark of mature astrocytes [35]. GFAP controls astrocyte motility and shape by providing structural stability to extensions of astrocytic processes but again the duration of the sleep deprivation episode is probably too short to elicit astrocyte proliferation. The observed increase in the abundance of GFAP in one spot may indicate an effect of sleep deprivation on cognitive function and synaptic plasticity. Indeed as both inhibitory avoidance and habituation alter the in vitro phosphorylation of GFAP [36], the observed change in abundance of GFAP in one spot might reflect such post-translational modification underlying a change in synaptic activity and/or efficacy induced by a stressful situation.

Dihydropyrimidinase-related protein 2 (ratio of -1.64) plays an important role in axon specification and elongation by regulating microtubule assembly, endocytosis of adhesion molecules, reorganization of actin filaments, and axonal protein trafficking [37]. In the adult brain, the expression of dihydropyrimidinase-related protein 2 is dramatically downregulated [38]. However, it remains expressed in structures that retain their capacity for differentiation and plasticity as well as in a subpopulation of oligodendrocytes. One hypothesis could be that this protein may be involved in neuron plasticity in the hippocampus during sleep deprivation.

Finally, since the transcriptional activator protein Pur-*α *has been implicated in diverse cellular functions, including transcriptional activation and repression, translation and cell growth [39], its increased abundance (ratio of -1.23) may indicate an increase in DNA transcription and translation, compatible with a general increase of cellular activity. This could indicate a genomic process beginning during the short sleep deprivation event possibly leading to broader DNA transcription in case the sleep deprivation duration increases.

### Identification of proteins in hippocampus of undisturbed rats

Interestingly, two proteins were more abundant in the hippocampus during sleep, as compared to total sleep deprivation. Protein disulfide isomerase A3 (ratio of +1.83) was found to be more expressed in the hippocampus of undisturbed than of sleep-deprived rat. Protein disulfide-isomerase A3 is expressed in higher amounts in ischemia (another form of stress) in heart [40]. It also modulates the redox state of the endoplasmic reticulum(ER), providing control of the ER Ca^2+ ^homeostasis [41]. But its two main roles are to catalyze disulfide bond formation and isomerisation in proteins and to inhibit their aggregation [42]. Disulfide bonds play a major role in the folding and stability of many proteins. As different authors suggested an involvement of sleep in protein synthesis [29], an increased protein disulfide isomerase A3 activity may be involved in the control of the proper folding of newly synthesized proteins. This suggestion deserves two qualifications. First, since the sleep episodes were short in our experiment, protein disulfide isomerase A3 could rather be involved in the control of the release of previously inactivated proteins, a process that could be faster than an activation and translation of genes related to sleep [9]. Second, we do not argue that sleep generally promotes protein translation, folding and degradation. Indeed, thiopurine S-methyltransferase (ratio of -1.42) also plays several roles in protein folding depending on its polymorphism and catalyzes the AdoMet-dependent S-methylation of thiopurine drugs such as 6-mercaptopurine [43] and is more abundant in the hippocampus after sleep deprivation.

Also, present in higher abundance in undisturbed rats, YPEL4 (ratio of 1.53) is a protein whose gene is highly conserved and expressed in various eukaryotic organisms [44]. This suggests that it must play important roles in the maintenance of life. Moreover, its subcellular localization (in association with centrosome or mitotic spindle) suggests a function in cell division. Unfortunately, its real function has not been uncovered yet.

### Sleep deprivation and the confounding effect of stress

The number of manipulations which were required to prevent the occurrence of sleep increased with the time spent in the experiment, demonstrating an increase of the sleep propensity. The rate of interventions reached a plateau after 3 hours, at the time when the rats' sleep propensity was at its highest [45]. This result is consistent with a previous study in which 3 hours of sleep deprivation triggers the homeostatic regulation of sleep, as indicated by subsequent episodes of sleep rebound [46].

An important issue for the interpretation of these results is to assess whether the higher abundance of some proteins is associated with wakefulness or rather reflects the consequences of stress. To address this issue, we characterized the effects of stress at two levels of description. First, at the macroscopic level, there was no evidence for sleep deprivation being stressful: we could not observe any stomach ulceration, any adrenal hypertrophy or any body weight loss in our rats. This could be expected as the stress duration was probably too short to induce the responses typically observed after 72 hours [47]. Second, at a molecular level, we characterized the protein patterns in adrenals of sleep-deprived rats.

### Identification of proteins in adrenals of undisturbed rats

Interestingly, we found more proteins with an increased abundance in the adrenals from rat left undisturbed as compared to adrenals of sleep-deprived rats, an opposite trend to what we found in the hippocampi. Most proteins identified in adrenals and more abundant in undisturbed rats belong to cell metabolism. They catalyze the transformation of carboxylic ester (carboxylesterase, ratio of +2.05), aldehyde (aldehyde dehydrogenase, ratios of +1.93 and +2.32), trimethylaminobutyraldehyde (aldehyde dehydrogenase family 9 subfamily A1, ratio of +1.79) and serine (serine hydroxymethyltransferase, ratio of +1.23). One protein is also specifically involved in the synthesis of ATP: NADH dehydrogenase 1 *α *(ratio of +1.71). This potential increase in ATP production can be advantageous for the increased activity in the metabolic pathways cited above.

Some identified proteins are involved in protein assembly and the regulation of transcription. In the mitochondrion, the steroidogenic acute regulatory protein (ratio of +1.24) enhances the transport of cholesterol and its metabolism into pregnenolone, a steroid hormone involved in steroidogenesis (of progesterone, androgens and estrogens a.o.) [48]. In the endoplasmic reticulum, the heat shock 70kDa protein 5 (ratio + 1.28) facilitates the assembly of multimeric protein complexes. And finally, in the cytoplasm, aspartyl t-RNA synthase (ratio of +1.31) assembles L-aspartyl and a t-RNA and consumes ATP. By getting involved in various metabolic steps and in various cell compartments, the increase in abundance of these proteins indicates an increase in protein transcription.

All these identified proteins contribute to the view of sleep as a mechanism of energy repletion [4] and restoration of molecular stocks [2].

Finally, serum albumin (ratio of +1.27), the most abundant blood plasma protein, can be considered here as an experimental artifact.

### Identification of proteins in adrenals of sleep-deprived rats

Another argument in favor of the absence of physiological stress due to sleep deprivation by gentle handling for 4 hours is the relatively low number of proteins the abundance of which increases in the adrenals of sleep-deprived rats. However one important protein identified here is glucose-6-phosphate dehydrogenase (ratio of -1.49). This enzyme catalyzes the first step of the oxidative phase of the pentose phosphate pathway. It is thus involved in the synthesis of NAPDH that may counteract oxidative stress generated following sleep-deprivation [49].

Stress can also be an important factor in the higher abundance of chloride intracellular channel protein 4 (ratio of -1.25) as chloride channels are important for maintaining a proper cell volume and cell resting membrane potential [50]. These two important factors can be modified by stress at a cellular level.

*α*-1-Macroglobulin (ratio of -1.27) is a widely expressed protein able to inhibit proteinases [51]. Its presence here could be explained by the fact that it is also an inhibitor of coagulation and could then be treated as an artifact rather than as a protein with a precise physiological function.

## Conclusion

By using a proteomic approach, this study has demonstrated that a short-term sleep deprivation affects various networks of proteins mainly related to cell metabolism, energy pathways, transport and vesicle trafficking, cytoskeleton and protein processing (translation, folding and degradation) in the hippocampus, a brain region central to cognition. The overall increase in abundance of these proteins in the sleep-deprived condition could indicate a general activation of a large number of cellular mechanisms involved in the maintenance of wakefulness, energy metabolism and maybe some cognitive function. The use of a CyDye immunoblotting method allowed us to quantitatively confirm the differences in abundances of several proteins, both in the hippocampus and the adrenals of rats.

Although we did not find any sign of behavioral stress, a difference in protein abundance was observed between sleep-deprived and undisturbed rat adrenals. This trend is in opposition to the one observed in hippocampi, with more proteins more abundant in the undisturbed condition compared to the sleep-deprived condition. This finding contributes to the view of sleep as a mechanism of energy repletion [4] and restoration of molecular stocks [2].

One should pay attention to two important points. First, our comparative approach detected not only proteins that are produced or degraded to higher levels after sleep or sleep-deprivation, but also proteins subjected to post-translational modifications, leading to a change in isoelectric point and/or molecular mass of their constituting subunits. Second, we only looked at the acidic part of the proteome. We were then unable to spot differences in the abundance of, for example, the early growth response protein (EGR1), a protein with an alkaline isoelectric point which is translated from a well-known experience-modulated gene [52].

An interesting question arising from these findings, to be explored by future research, is how sleep deprivation interacts at a molecular level with cognitive processes since sleep deprivation disrupts the processing of recent memory traces [53] and extends the effects of synaptic potentiation on subsequent homeostatic processes [21]. It will also be interesting to look at the differences in proteins abundances at different time points in the sleep deprivation.

## Methods

### Animals

Male Sprague-Dawley rats weighing 170–220 g at the start of the experiment were housed by pairs and maintained in a 12-h light-dark cycle with lights on at 08:00, with standard food (PLUS type, SAFE) and water available ad libitum. Rats were accustomed to the housing facilities for at least 7 days prior to the beginning of any experiments. All experiments were approved by the Animal Ethical Committee of the University of Liege.

### Sleep-deprivation and alertness state monitoring

In the first group, rats (n = 9) were sleep-deprived by gentle handling [54]. The animals were continuously observed during 4 hours at the beginning of the light period. Sleep deprivation was achieved by disturbing the cage bedding around the rat, stroking the vibrissae using a small brush and gently stroking the fur with the brush when the rat assumes a sleep posture [55]. In the second group, rats (n = 9) were left undisturbed during these 4 hours.

Alertness state was monitored by two means: an automated activity recording setup and program, Gemvid [56], developed in our laboratory, and manual behavioral scoring. For each rat, the alertness level was determined during the light period before the test. Scores below this level during the test were considered as rest/sleep. Rat behavior was also manually scored by selecting alertness states as soon as they occured with software developed in our laboratory. The following alertness states were defined: rest/sleep, movement, eating, washing, foraging, and environment exploration. The software allowed the quantification of the number and duration of rest/sleep periods as well as the number and frequency of manual awakenings for sleep-deprived rats. Statistical analyses (ANOVA, Tukey and Kruskal-Wallis methods) were performed in Statistica 7.1 (Statsoft).

All rats were weighed before and after the observation.

### Adrenals, hippocampus and protein extraction

Rats were decapitated immediately after the 4 hour period of sleep or sleep deprivation, and the brains were quickly removed and dissected on ice. The hippocampi were extracted from the brain according to Hortnagl et al. [57], weighed and quickly frozen at -80°C. The rat thoracic cavity was opened, the viscera were removed and adrenals were cut, weighed and quickly frozen at -80°C.

Proteins were extracted by 10 strokes of a Potter homogenizer in a lysis buffer containing 7 M urea (ICN), 2 M thiourea (GE Healthcare), 30 mM Tris at pH 8.5, and 2% ASB-14 (Sigma). The supernatant containing the solubilised proteins was precipitated using the 2-D Clean-Up Kit (GE Healthcare) and protein amounts were determined with the RC DC Protein Assay (Bio-Rad).

The stomach was cut along the smaller curvature, cleaned, pinned on an inspection board and examined for ulcerations.

### Protein labeling and 2-D Differential in-gel electrophoresis

CyDyes minimal labeling was performed according to the procedure published by Tonge et al. [58] with minor modifications. Briefly, each 25 *μ*g of sample protein was mixed with 200 pmol of CyDyes (GE Healthcare) reconstituted in anhydrous dimethylformamide (Aldrich), according to its labeling group, and left for 30 minutes in the dark, at +4°C. The coupling reaction was then stopped by adding the same volume of 10 mM lysine (Acros) for 10 minutes at +4°C. An internal standard was prepared by mixing equal amounts of all the samples within the experiment. Experimental samples were either labeled with Cy3 or Cy5. Then they were mixed in pairs together with 25 *μ*g of the internal standard labeled with Cy2. For the first dimension (isoelectric focusing), a rehydration buffer was added to each analytical sample. For preparative gels, the rehydration buffer was added to 250 *μ*g of unlabelled proteins. This buffer contained 7 M urea, 2 M thiourea, 2% ASB-14, 0.2% dithiothreitol and 0.5% IPG buffer 4–7 (GE Healthcare). The solution was then poured in a strip holder, covered with a pH 4–7 IPG strip (GE Healthcare) and finally covered with 1 ml PlusOne DryStrip cover fluid (GE Healthcare). The following electrophoresis steps were successively applied: 12 hours of rehydration, 1 hour at 500V (step-and-hold), 1 hour to 1000V (gradient), 3 hours to 8000V (gradient) and 5.5 hours at 8000V (step-and-hold).

Before initiating the second dimension step, proteins in IPG strips were reduced in an equilibration solution (50 mM Tris-HCl, pH 8.8, 6 M urea, 30% glycerol (Bio-Rad) et 1.6% SDS (MP Biomedicals)) containing 1% DTT. They were then alkylated in the same equilibration solution containing 5% iodoacetamide (GE Healthcare).

IPG strips were placed on top of a classical SDS-PAGE gel (12.5% acrylamide (GE Healthcare)).

Electrophoretic migration was performed in an Ettan Dalt six apparatus (GE Healthcare) at 2.5 W per gel during 30 minutes and then 100 W for a maximum of 4 hours.

Once the second dimension step was realized, each gel was scanned at three different wavelengths corresponding to the different CyDyes with a Typhoon 9400 Laser Scanner (GE Healthcare). Gel images were cropped with the ImageQuant 5.2 program (Molecular Dynamics) in such a way that all the images represented the same area.

### Spot detection and pattern analysis

After scanning the gels, 2-D gel analysis software (DeCyder version 6.5, GE Healthcare) was used in this study for spot detection, gel matching and spot quantification relative to the corresponding spot in the internal standard [59]. After matching, spots showing a statistically significant variation in relative abundance of at least 1.2 fold (Student t test, p < 0.05) and a minimal volume of 250,000 were selected for further analysis (Biological Variation Analysis (BVA) module of Decyder software). All spots considered by the software as significantly regulated at a threshold of ± 1.2 were visually checked, possibly selected and a pick list was created containing all spots with proteins sent to mass spectrometry.

### Spot excision, digestion and identification of proteins

All differentially expressed spots were automatically excised from the gels with an Ettan Spot Picker robot (GE Healthcare). The picker head had a diameter of 2.0 mm. Gel pieces were collected in 96-well plates designed for the digestion of proteins.

For hippocampal protein digestion, gel pieces are collected in 96-well plates designed for the Proteineer dp automated digester (Bruker Daltonics). Briefly: gels pieces are washed with 3 cycles of successive soaking in 100% ammonium hydrogenocarbonate 50 mM and a mix of 50% acetonitrile/50% ammonium hydrogenocarbonate 50 mM. Two additional washes are performed with 100% acetonitrile to dehydrate the gel pieces. 3 *μ*l of freshly activated trypsin (Roche, bovine, sequencing grade) 10 ng/*μ*l in ammonium hydrogenocarbonate is used to rehydrate the gel pieces at +8°C for 30 minutes. Trypsin digestion is performed for 3 h at 30°C. Peptide extraction is performed with 10 *μ*l of 1% formic acid for 30 minutes at 20°C. Hippocampal protein digests (3 *μ*l) are then adsorbed for 3 minutes on prespotted anchorchips (Bruker). Spots are washed on-target using 10 mM dihydrogeno-ammonium phosphate in 0.1% TFA-MilliQ water to remove salts. High throughput spectra acquisition is performed using an Ultraflex II MALDI mass spectrometer (Bruker) in positive reflectron mode, with close calibration enabled, Smartbeam laser focus set to medium, and a laser fluency setting of 65 to 72% of the maximum. Delayed extraction is set to 30 ns. Steps of 100 spectra in the range of 860 to 3800 Da are acquired at a 200 Hz LASER shot frequency with automated evaluation of intensity, resolution and mass range. 600 successful spectra per sample are summed, treated and de-isotoped in line with an automated SNAP algorithm using Flex Analysis 2.4 software (Bruker), and subsequently submitted in the batch mode of the Biotools 3.0 software suite (Bruker) with an in-house hosted Mascot search engine (MatrixScience.com) to the NCBI rodent database. The Swiss-Prot 50.5 release and NCBInr_20060605 release databases are used for the rat species. A mass tolerance of 100 ppm with close calibration and one missing cleavage site are allowed. Partial oxidation of methionine residues and complete carbamylation of cystein residues are considered. The probability score calculated by the software was used as a primary criterion for correct identification. For mascot scores slightly above the threshold of p value, measurement errors were carefully watched to detect possible false positive that usually give random mass measurement errors, while relevant identifications give constant measurement errors. Experimental and Mascot results molecular weights and pI were also compared. Adrenal protein digestion and spotting on Maldi targets have been carried out as previously described in Bohler et al. [60]. Peptide mass determinations (PMF and MS/MS) were carried out using the Applied Biosystems 4800 Proteomics Analyzer (Applied Biosystems). Calibration was performed with the peptide mass calibration kit for 4700 (Applied Biosystems). Proteins were identified by searching against an in-house database downloaded from NCBi and by using Mascot (MatrixScience.com). The parameters for search in database allowed 2 missed cleavages, a tolerance of 0.5 Da on MS/MS fragments and 100 ppm on precursor mass, as well as carbamidomethylation on cysteine and oxidation of methionine as fixed modifications. For both identification, the probability score (Mowse score [61]) calculated by the software was used as a criterion for correct identification.

### Western blots

To confirm the presence of some proteins and the difference between abundance levels, 1D western blots were performed with the same protein samples used in the proteomic analysis. *α*-SNAP and NSF were detected in the hippocampal protein extracts with mouse monoclonal antibodies (ab16391, concentration 1 *μ*g/ml; ab16681, dilution 1/2000, Abcam). Glucose-6-phosphate dehydrogenase in the adrenals was detected with a rabbit polyclonal antibody (ab993, dilution 1/1000, Abcam).

For western blots, 50 *μ*g of proteins was combined with an equal volume of Laemmli buffer and separated by electrophoresis performed on a 12% SDS-PAGE with a 4% stacking gel (in a Mini Trans-Blot Cell, Bio-Rad). A molecular weight ladder was separated along with the proteins of interest (MagicMark, Invitrogen). Proteins in the gel were then electrophoretically transferred (Trans-Blot SD Semi-Dry Transfer Cell) to a 0.2-*μ*m low-fluorescent PVDF membrane (Hybond-LFP, Amersham). The membrane was blocked with 3% nonfat dry milk in Tween-Tris-buffered saline (TTBS) for 1 h at room temperature and incubated with the corresponding primary antibody in TTBS with 3% dry milk overnight at +4°C with gentle shaking. The membrane was washed with TTBS three times (10 minutes each), incubated in either ECL Plex goat-anti-mouse IgG Cy3 or goat-anti-rabbit Cy5-conjugated secondary antibody (dilution 1/2500, GE Healthcare) for 1 hour at room temperature and washed again three times in TTBS (15 minutes each). Membranes were then washed twice in TBS (15 minutes each). After drying at +37°C, membranes were scanned at the corresponding wavelengths with a Typhoon 9400 Laser Scanner (GE Healthcare). Membrane images were analyzed with the ImageMaster 1D software (GE Healthcare).

## Authors' contributions

JEP designed and conceived the study, carried out the behavioral and proteomics experiments and drafted the manuscript. FG carried out part of the identification of hippocampal proteins. JR and KS carried out the identification of adrenal proteins. AL and PM participated in the design and coordination of the study. PL conceived the study and participated in its design and coordination. All authors helped to draft the manuscript. All authors read and approved the final manuscript. The authors declare that they have no competing interests.
